# Immune Responses in the Central Nervous System Are Anatomically Segregated in a Non-Human Primate Model of Human Immunodeficiency Virus Infection

**DOI:** 10.3389/fimmu.2017.00361

**Published:** 2017-03-30

**Authors:** Barbara Tavano, Vicky Tsipouri, Gareth A. D. Hardy, Caroline M. Royle, Michael R. Keegan, Dietmar Fuchs, Steven Patterson, Neil Almond, Neil Berry, Claire Ham, Deborah Ferguson, Adriano Boasso

**Affiliations:** ^1^Centre for Immunology and Vaccinology (CIV), Imperial College London, Chelsea and Westminster Hospital, London, UK; ^2^NIHR Biological Research Unit, Royal Brompton Hospital, London, UK; ^3^ViiV Healthcare, Middlesex, UK; ^4^Division of Biological Chemistry, Biocenter, Innsbruck Medical University, Innsbruck, Austria; ^5^Division of Virology, National Institute for Biological Standards and Controls (NIBSC), Potters Bar, Hertfordshire, UK

**Keywords:** simian immunodeficiency virus, central nervous system, neuroinflammation, neurotoxicity, indoleamine (2,3)-dioxygenase

## Abstract

The human immunodeficiency virus (HIV) accesses the central nervous system (CNS) early during infection, leading to HIV-associated cognitive impairment and establishment of a viral reservoir. Here, we describe a dichotomy in inflammatory responses in different CNS regions in simian immunodeficiency virus (SIV)-infected macaques, a model for HIV infection. We found increased expression of inflammatory genes and perivascular leukocyte infiltration in the midbrain of SIV-infected macaques. Conversely, the frontal lobe showed downregulation of inflammatory genes associated with interferon-γ and interleukin-6 pathways, and absence of perivascular cuffing. These immunologic alterations were not accompanied by differences in SIV transcriptional activity within the tissue. Altered expression of genes associated with neurotoxicity was observed in both midbrain and frontal lobe. The segregation of inflammatory responses to specific regions of the CNS may both account for HIV-associated neurological symptoms and constitute a critical hurdle for HIV eradication by shielding the CNS viral reservoir from antiviral immunity.

## Introduction

Infection by the human immunodeficiency virus (HIV) type 1 causes a progressive impairment of the immune system, characterized by systemic immune activation and functional exhaustion which, if left untreated, lead to the fatal acquired immunodeficiency syndrome (AIDS) (1). Before the advent of antiretroviral therapy (ART), between 20 and 30% of HIV-infected patients developed HIV-associated dementia (HAD), which usually presented as a severe motor and cognitive disorder (2), and HIV-associated encephalitis during the late stages of infection (3, 4). Astrogliosis, microglial activation, and accumulation of perivascular macrophages characterize the immune phenotype in the central nervous system (CNS) during HIV infection (5, 6).

Antiretroviral therapy can effectively control HIV replication in the majority of patients (7). However, underlying systemic immune activation can give rise to tissue-specific comorbidities associated with localized inflammation (8). In particular, even in the ART era, approximately 20–40% of patients experience HIV-associated neurocognitive disorder (HAND) (9, 10). The clinical manifestations of HAND are milder than those observed in HAD and are characterized by mild disturbances of psychomotor speed, processing speed, executive function, or memory (11). Albeit milder than severe pre-ART era HAD, the neurological symptoms typical of HAND still affect daily function, ART adherence, and quality of life (11, 12). Residual viral replication due to incomplete penetration of antiretrovirals in the CNS, and the ensuing inflammatory response, could both contribute to the neurological symptoms during HIV infection (3, 4). In addition, some antiretroviral drugs may have neurotoxic effects (13).

Human immunodeficiency virus is thought to gain access to the CNS early during infection, probably within infected macrophages which cross the blood–brain barrier (BBB) (14, 15). HIV DNA has been detected in infiltrating macrophages, microglial cells, and astrocytes in CNS tissue samples collected postmortem from HIV-infected patients, even in the absence of neurological symptoms (16, 17). The unique population of target cells for infection in the CNS promotes viral compartmentalization and independent evolution of sub-quasispecies of HIV with enhanced neurotropism (18–22).

Activated T lymphocytes and macrophages have the ability to cross the BBB and infiltrate the CNS (23–25). However, it is still unknown whether peripheral lymphocytes reach or are attracted to sites of HIV infection within the CNS and therefore contribute to control viral replication. The half-life of macrophages and microglial cells, ranging from months to several years (26, 27), combined with the immunologic characteristics of the CNS, raise the possibility that a sanctuary for latent HIV reservoirs is established in this anatomical locale, which may represent a major hurdle for HIV eradication (28, 29).

Infection of non-human primates with different strains of simian immunodeficiency virus (SIV) has been utilized to mimic HIV-induced neuropathology (30–33). However, highly neurotropic SIV strains may produce symptoms similar to severe forms of HAD, which are not representative of the low level neuroinflammation that occurs in the majority of HIV-infected patients (34). Fewer data are available on low grade neuroinflammation and viral replication during the normal course of infection, and on how the inflammatory responses diverge in different regions of the CNS.

Simian immunodeficiency virus infection in cynomolgus macaques may offer a platform to study CNS inflammation in an animal model that more closely resembles the pathogenesis of human HIV-1 infection compared to rhesus macaques and highly neuropathogenic models of infection. Thus, SIV-infected cynomolgus macaques exhibit lower viremia at both peak and set point, resulting in slower progression to disease with more typical AIDS defining illnesses (33, 35, 36).

Here, we assessed whether differences in inflammatory responses exist among anatomical sites in the CNS during SIV infection. We analyzed gene expression in brain explants from midbrain, frontal lobe, and cerebellum of macaques, which were challenged with low dose SIV and showed transient or persistent viremia, compared with those that remained uninfected. Our data indicate that inflammatory responses differ dramatically between midbrain and frontal lobe, a dichotomy which is not associated with measurable differences in viral transcriptional activity.

Understanding the dynamics of the inflammatory responses in the CNS during HIV infection may provide critical information on both the pathogenesis of HIV-associated cognitive impairment, and the potential for immunotherapeutic approaches to clear the latent viral reservoirs.

## Materials and Methods

### Animal Ethics Statement

Non-human primates were used in strict accordance with UK Home Office guidelines, under a license granted by the Secretary of State for the Home Office. NIBSC is governed by the Animals (Scientific Procedures) Act 1986, which complies with the EC Directive 86/609 and performs under license (PPL 80/1952) granted only after review of all license procedures by the NIBSC Ethical Review Process. All macaques were purpose bred and group housed for the study duration, with daily feeding and access to water *ad libitum*. Regular modifications to the housing area were made by husbandry staff to further enrich the study environment. Animals were acclimatized to their environment and deemed healthy by the named veterinary surgeon prior to study inclusion.

All animals were sedated prior to bleeding or virus inoculation by venipuncture. Frequent checks were made and unexpected changes in behavior followed up, including seeking of veterinary advice where necessary. Regular blood samples were obtained to assess hematological parameters that might provide evidence of incipient disease and veterinary advice sought when persisting abnormalities detected. The study was terminated and all animals killed humanely by administering an overdose of ketamine anesthetic prior to development of overt symptomatic disease. All efforts were made to minimize animal suffering, including the absence of procedures not essential to the study.

### Animals and Viral Challenges

Twelve purpose bred juvenile, simian retrovirus negative, and simian T lymphotropic virus negative cynomolgus macaques were used in these studies. Macaques K4, K6, K7, K9, K10, and K11 were vaccinated against full length SIVmac239 gag before being subjected to viral challenges. Specifically, they received three 100 µg DNA intradermal inoculations 23, 19, and 15 weeks before viral challenge, and a boost with replication-defective adenoviruses 5 vector 6 weeks before challenge. Animals K20, K21, K23, K24, K25, and K26 were the unvaccinated control group. All animals were challenged intrarectally with 150TCID_50_ SIVmac251 starting at week 1 and for up to 10 weeks. Blood samples were taken weekly and tested for plasma SIV RNA by PCR, as described below. Following a positive PCR result, animals were no longer challenged. Animals were euthanized 19–20 weeks after study.

The implications that the vaccination protocol may have had for the data on neuroinflammatory responses reported in this study are considered in both the Sections “Results” and “Discussion.”

### Plasma Viral Load Measurement

Simian immunodeficiency virus viral RNA loads were determined in plasma samples collected at various times throughout the course of the study by quantitative real-time reverse transcriptase PCR, as previously described (37).

### Collection of Brains and Tissue Preparation

Whole brains were harvested no longer than 1 h post-termination. Tissue explants of approximately 50–100 mg were collected from the frontal lobe, midbrain, and cerebellum, immersed in RNA*later* (Qiagen, Manchester, UK) and stored at −80°C until RNA extraction. Whole brains were then fixed in 10% (v/v) formal saline for 4 weeks at 4°C, dissected into defined brain regions and processed for paraffin embedding (33). Following dewaxing, 4 µm formalin-fixed paraffin embedded brain sections were stained with hematoxylin in accordance with standard histochemical techniques.

Because the animals were originally included in a study aimed at assessing the efficacy of a vaccination protocol, fresh tissue, and blood samples had to be collected at termination. The multiple breaks introduced during removal of tissues meant that the vascular system could not support the flushing of saline and then formalin. Thus, none of the animals had undergone perfusion prior to brain collection. The implications that residual leukocytes or viral particles within the blood vessels may have had on our findings are considered in the Sections “Results” and “Discussion.”

### RNA Extraction and Reverse Transcription

Total RNA was extracted from RNA*later*-preserved explants using the RNeasy Midi Kit (Qiagen), according to the manufacturer’s instructions. Samples were thawed at room temperature, removed from RNA*later*, and placed in the lysis buffer provided with the RNeasy Midi Kit. Tissue homogenization was performed using a Precellys Minilys bead beater homogenizer (Bertin Technologies, France) in tubes of 2 ml capacity with 1.4 mm ceramic (zirconium oxide) beads (Precellys soft tissue homogenizing kit, CK14). Total RNA was eluted in molecular grade RNase/DNase-free water (Qiagen), yield and quality were tested by absorbance measurement at 260, 280, and 230 nm using a NanoDrop™ Lite Spectrophotometer (Thermo Scientific, UK), and samples were stored at −20°C until use. Reverse transcription was carried out using the RT^2^ First Strand Kit (Qiagen), according to manufacturer’s instructions. cDNA samples were stored at −20°C until use in the real-time PCR arrays. This same procedure was used for both the multigene arrays and for the additional single gene real-time PCR described below.

### Multigene Real-time PCR Arrays

Two targeted real-time PCR gene arrays were performed on cDNA from SIV-macaques brain tissue samples: (1) an array measuring the expression of 84 genes coding for inflammatory cytokines, chemokines, and their receptors (RT^2^ profiler gene array PAQQ-011Z, Qiagen) was used on cDNA samples from frontal lobe, midbrain, and cerebellum; and (2) an array measuring the expression of 84 genes that are either upregulated or downregulated during processes associated with neurotoxicity (RT^2^ profiler gene array PAQQ-069Z, Qiagen) was used on cDNA samples from frontal lobe and midbrain. Full lists of the genes included in each array are given in Tables S1 and S2 in Supplementary Material. Arrays were performed according to the manufacturer’s instructions on a LightCycler-480 (Roche Diagnostics Ltd., Burgess Hill, UK) with 96-well plate block. Briefly, the reaction plates are already seeded with appropriate primers set, including five housekeeping genes, one genomic DNA control, triplicate RT, and PCR positive controls. A reaction mix containing sample cDNA and all reagents required for a SYBR green-based detection system, with the exception of the primers, was prepared using the RT^2^ qPCR Master Mixes provided with the kit and dispensed in each well. Reactions were performed according to the following thermal profile: initial denaturation at 95°C for 10 min; 45 cycles of 95°C for 10 s and 60°C for 1 min, fluorescence data collection performed at the end of the 60°C step.

Initial data analysis was performed using the LightCycler-480 operating software for automatic estimation of background noise and fluorescence threshold. Data were normalized on the average of at least three housekeeping genes, which showed a coefficient of variability (CV = SD/mean) lower than 10% across all tissues and among all animals within the same array. Specifically, data from the PAQQ-011Z array were normalized on the average of three housekeeping genes (ACTB, CV = 9.76%; B2M, CV = 8.06%; GAPDH, CV = 9.03%; combined CV = 8.02%); data from the PAQQ-069Z were normalized on the average of all five housekeeping genes (ACTB, CV = 4.77%; B2M, CV = 6.35%; GAPDH, CV = 3.23%; HPRT1, CV = 4.51%; RPL13A, CV = 5.39%; combined CV = 4.41%). RT and PCR positive controls were used to calculate inter-plate variability (PAQQ-011Z: RT CV = 0.79%, PCR CV = 1.06%; PAQQ-069Z: RT CV = 0.29%, PCR CV = 0.29%).

### Real-time PCR for Immunoregulatory Molecules and SIVgag

The expression of genes encoding for interleukin (IL)-10, cytotoxic T lymphocyte antigen 4 (CTLA-4), the forkhead family transcriptional regulator FOXP3, transforming growth factor (TGF)-β, and the SIV capsid protein gag was tested by SYBR green-based real-time PCR on cDNA from brain explants collected as described above. All reactions were performed in a LightCycler-480 (Roche) using SYBR green PCR mastermix (Qiagen); a list of primers used for each target and the housekeeping gene GAPDH is provided in Table S3 in Supplementary Material. All reactions were performed according to the following thermal profile: initial denaturation at 95°C for 10 min; 40 cycles of 95°C for 20 s, 60°C for 20 s, and 72°C for 20 s (fluorescence data collection performed at the end of the 72°C step).

### Kynurenine and Tryptophan Measurement

Cerebrospinal fluid (CSF) was collected at necropsy from all animals with the exception of K10. CSF concentrations of TRP and KYN were measured by high performance liquid chromatography as previously described (38).

### Statistical Analysis

Statistical analysis was performed using R version x64 3.2.2 (The R Foundation for Statistical Computing) and SPSS version 22 (IBM, Armonk, NY, USA). Microsoft Excel 2013 (Microsoft Corporation, Redmond, WA, USA) and GraphPad Prism version 5 (GraphPad Software, La Jolla, CA, USA) were used to generate some of the plots in the figures. Comparison between uninfected and SIV-infected animal groups was performed using non-parametric Mann–Whitney test. Due to the small sample size, the results obtained in this study did not survive stringent false discovery rate controlling procedures. Therefore, only genes showing at least 1.5-fold difference between the two groups, calculated as the ratio between median values, and uncorrected *P* < 0.05 were considered to be upregulated or downregulated. All correlations were assessed using non-parametric Spearman test.

## Results

### Infection Rate and Viremia Levels Following Repeated Low Dose SIVmac251 Challenge

**Figure 1:**
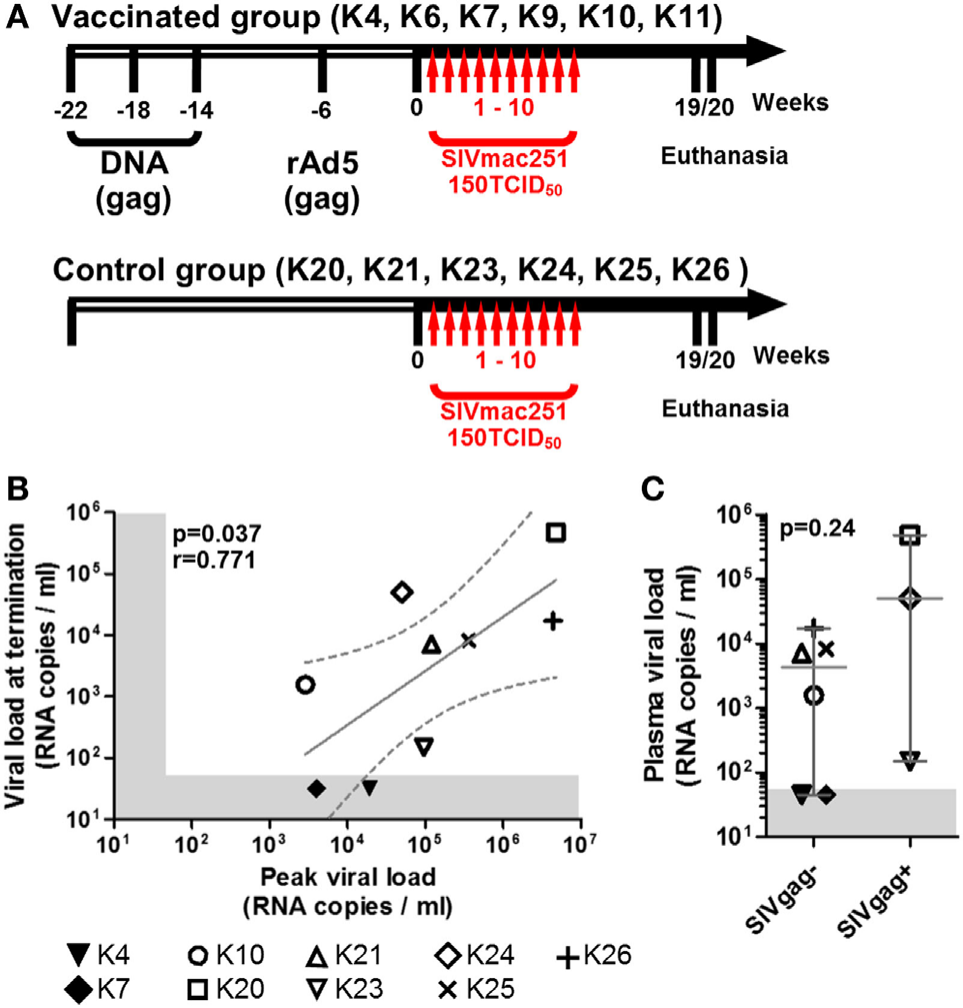
**Macaques history, challenge schedule, and plasma viral loads**. **(A)** Schematic representation of macaques treatment before (empty side of the arrow) and after inclusion in the study (solid black side of the arrow); vertical red arrows indicate weekly SIV challenges. **(B)** Scatter plot showing the direct correlation between peak of viremia (highest level of viremia measured throughout the study) and the viremia at study termination of all SIV+ animals (non-parametric Spearman correlation); solid gray line shows the projected linear regression, and dotted lines show the 95% confidence interval. **(C)** Dot plot showing the difference in plasma viremia at termination between animals with detectable and undetectable SIVgag RNA in either midbrain or frontal lobe explants (SIVgag+ and SIVgag−, respectively); statistical analysis was performed using a non-parametric Mann–Whitney test. In both plots, shaded areas indicate the range of undetectable viremia (<50 RNA copies/ml).

Twelve cynomolgus macaques were included in the study. All animals were challenged intrarectally with 150 TCID_50_ SIVmac251 at weekly intervals starting at week 1 for up to 10 weeks or until they tested positive for plasma SIV-RNA and were euthanized at week 19 or 20 (Figure 1A). All animals were originally included in a prophylactic vaccination study, schedule of vaccination and viral challenge is shown in Figure 1A. Eight of the twelve macaques showed positive plasma SIV RNA measurement by week 5 and one (K24) tested positive at week 11, after the last challenge (Table 1). Two of the nine infected animals (K4 and K7) had undetectable viremia at the time of necropsy and will be referred to as aviraemic (Table 1). Animals showing detectable plasma SIV RNA at necropsy (median 8.2 × 10^3^ SIV RNA copies/ml, range 1.5 × 0^2^–5.1 × 10^5^ SIV RNA copies/ml) will be referred to as viremic (Table 1). Peak of viremia generally occurred on the second week in which plasma tested positive for SIV RNA, with the exception of animals K10 (peak on the first positive sample) and K26 (peak on the third positive sample). A direct correlation was observed between peak of viremia and viremia measured at termination (Figure 1B). Three animals never showed detectable viremia throughout the study (K6, K9, and K11) and will be referred as uninfected (Table 1).

**Table 1:** **Virological information for the animals in the study**.

Group	Animal	First positive viremia	Peak viremia	Viremia at termination
Uninfected	K6	–	<50	<50
	K9	–	<50	<50
	K11	–	<50	<50
SIV+ aviraemic	K4	Week 4	19,200	<50
	K7	Week 3	4,010	<50
SIV+ viremic	K10	Week 5	2,880	1,560
	K20	Week 4	4,740,000	480,000
	K21	Week 4	120,000	7,100
	K23	Week 3	95,500	149
	K24	Week 11	50,300	50,300
	K25	Week 4	361,000	8,210
	K26	Week 2	4,390,000	17,200

All uninfected (K6, K9, and K11) and SIV+ aviraemic animals (K4 and K7) belonged to the vaccinated group (Figure 1; Table 1), whereas only animal K10 among the viremic group had previously received prophylactic vaccination. These observations suggest that the vaccination protocol employed in the study may have exerted a protective effect on infection or favored control of viral load in the periphery.

### SIV RNA Activity in the CNS

SIVgag RNA was detected only in the midbrain of animal K24 and frontal lobe of animals K20 and K23 and was undetectable in all other tissue explants. Because the animals were not perfused before collection of the brains, we cannot exclude the possibility that part of the detected viral RNA derived from viral particles trapped in blood vessels, rather than from virus produced *in situ*. Nonetheless, the vast majority of the explants tested negative for viral RNA. It is noteworthy that macaque K23, which tested positive for SIVgag RNA in the frontal lobe, displayed the lowest detectable plasma viral load at termination among viremic animals (Figure 1C).

### Expression of Inflammatory Genes in the CNS

The expression of 84 genes associated with inflammatory responses was measured by multigene real-time PCR array in homogenized brain explants collected postmortem from midbrain, frontal lobe, and cerebellum from *N* = 12, *N* = 11, and *N* = 11 macaques, respectively (frontal lobe and cerebellum explants were not collected from animal K4), and levels of gene expression were compared between uninfected (SIV−) and SIV+ animals.

**Figure 2:**
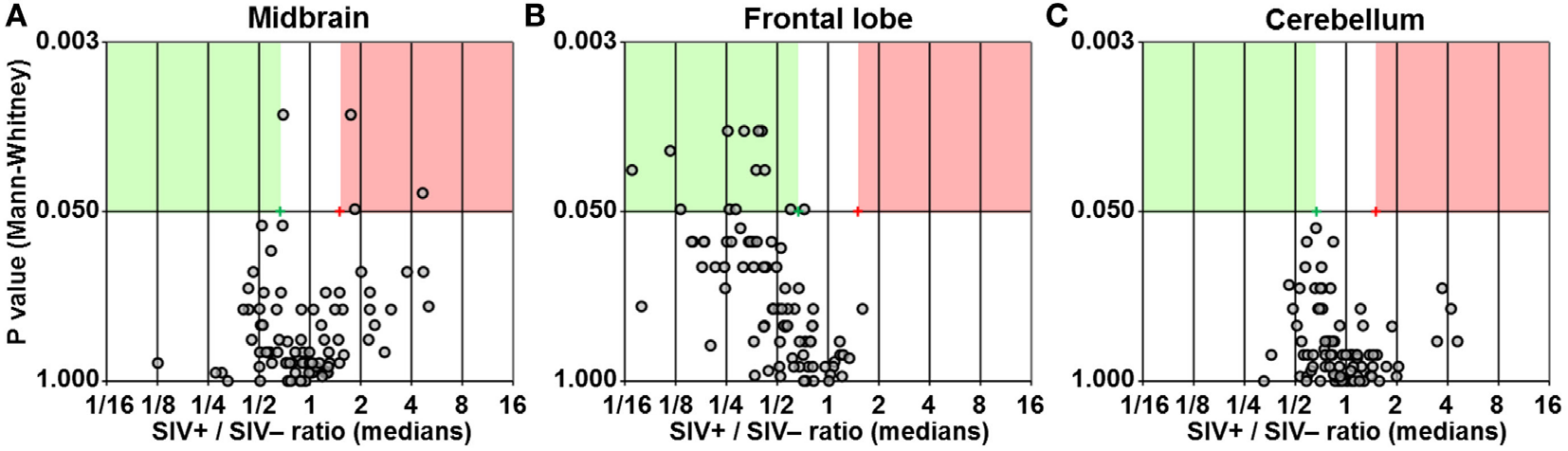
**Differential expression of inflammatory genes**. Volcano plots showing the ratio between median gene expression in simian immunodeficiency virus (SIV)-infected macaques and -uninfected macaques (*x* axis; Log_2_ scale), in relation to the *P* value (Mann–Whitney test; *y* axis; Log_20_ scale), for midbrain **(A)**, frontal lobe **(B)**, and cerebellum **(C)**; each dot represents one gene. Red and green areas highlight genes upregulated or downregulated in SIV-infected compared to -uninfected animals (1.5-fold; *P* < 0.05).

**Figure 3:**
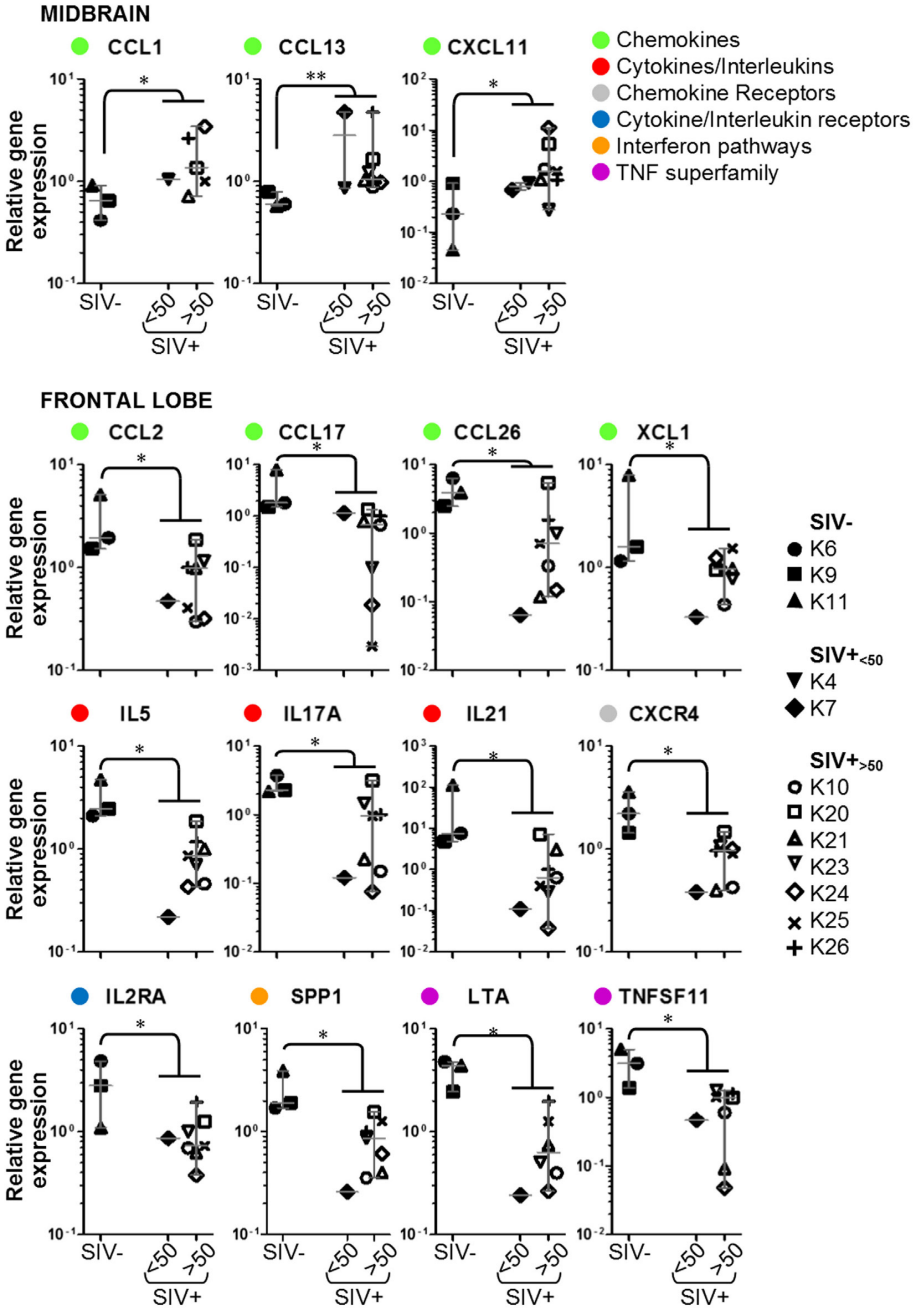
**Details of inflammatory genes that are upregulated or downregulated in simian immunodeficiency virus (SIV)-infected macaques**. Dot plots showing relative expression of the genes in the red and green areas of Figure 2 for uninfected (SIV−), SIV-infected aviraemic (SIV+_<50_), and viremic (SIV+_>50_) animals. All data are normalized against the median for all 12 animals. Each symbol indicates one animal as detailed in the legend; the legend refers to data from both midbrain and frontal lobe; horizontal gray lines within graphs indicate medians, and vertical gray lines show the interquartile ranges (*P < 0.05; **P < 0.01).

We found three genes upregulated by at least 1.5-fold with *P* < 0.05 in the midbrain of SIV-infected macaques (Figure 2A). Surprisingly, the same analysis in the frontal lobe showed 12 genes to be downregulated by at least 1.5-fold (*P* < 0.05) in SIV-infected compared to -uninfected animals (Figure 2B). No differences in any of the genes analyzed were observed in the cerebellum (Figure 2C). The expression profiles in both midbrain and frontal lobe of SIV-infected aviraemic animals had greater similarity to that of viremic rather than uninfected animals (Figure 3), suggesting that inflammatory responses in the CNS ensue early during infection, and are not affected by the level of ongoing viral replication. An alternative, non-mutually exclusive explanation is that plasma viremia is not a reliable indicator of viral replication in other anatomical compartments, including the CNS.

We considered whether the protective effect of the prophylactic vaccine had any influence on the expression of inflammatory genes in the brain. Focusing on the 15 genes that were differentially regulated between SIV− and SIV+ animals, we compared the expression levels in aviraemic animals that had received the vaccine (K4 and K7) to that observed in SIV+ viremic animals (SIV+_<50_ vs. SIV+_>50_ in all plots in Figure 3). Although stringent statistical analyses could not be performed due to the limited number of aviraemic animals, the levels of gene expression observed in animals K4 and K7 closely resemble those observed in viremic SIV+ animals. In addition, when we repeated the analyses shown in Figure 3 on a dataset that excluded animals K4 and K7, 10/15 of the parameters tested remained significantly different (*P* < 0.05) between SIV− and SIV+ animals, and the *P* values for the other 5/10 genes remained <0.1. These observations suggest that even if prophylactic vaccination may have allowed control of viral replication in the periphery, it did not prevent the immune alterations detected in the CNS.

We tested whether the downregulation of inflammatory genes in the frontal lobe was associated with differences in the expression of immunoregulatory genes encoding for interleukin (IL)-10, CTLA-4, the forkhead family transcriptional regulator FOXP3 or TGF-β. Expression of all immunoregulatory genes was below detection level in at least 80% of the samples tested, with the exception of TGF-β, which, however, did not differ between SIV-infected and -uninfected animals (data not shown).

### Tryptophan Catabolism *via* the Kynurenine Pathway in the CNS

**Figure 4:**
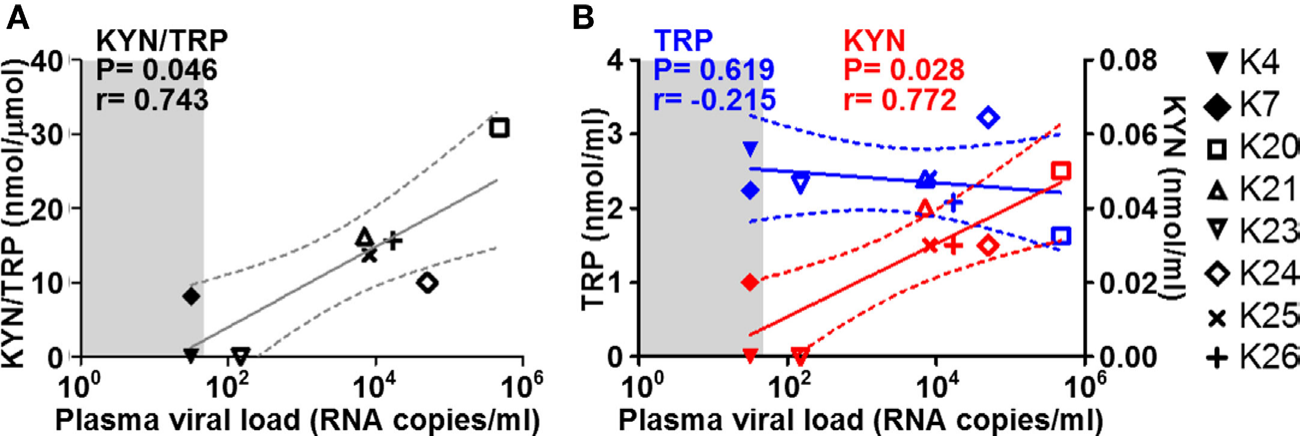
**Cerebrospinal fluid (CSF) KYN and TRP in relation to plasma viremia**. Scatter plots showing the correlations (non-parametric Spearman test) between plasma viremia at the time of necropsy (*x* axes) and **(A)** KYN/TRP ratios, **(B)** KYN (red), and TRP (blue) levels in the CSF. Solid lines show the projected linear regressions, and dotted lines show the 95% confidence intervals. Shaded areas indicate the range of undetectable viremia (<50 RNA copies/ml).

Increased tryptophan (TRP) catabolism by indoleamine 2,3-dioxygenase 1 (IDO1) has been suggested to contribute to inflammation-mediated neuronal damage by leading to the production of neurotoxic metabolites such as quinolinic acid (QA) (39). We measured the levels of TRP and its key metabolite kynurenine (KYN) in CSF collected at necropsy from the macaques in the study. The KYN/TRP ratio, a well-accepted measure for IDO1 activity, correlated directly with plasma viremia at termination in the SIV-infected group (Figure 4A). This correlation was mainly driven by alterations of KYN levels, rather than TRP (Figure 4B), suggesting an increase in catabolism at either peripheral or central level, rather than systemic depletion of TRP.

### Expression of Neurotoxicity Genes in CNS

**Figure 5:**
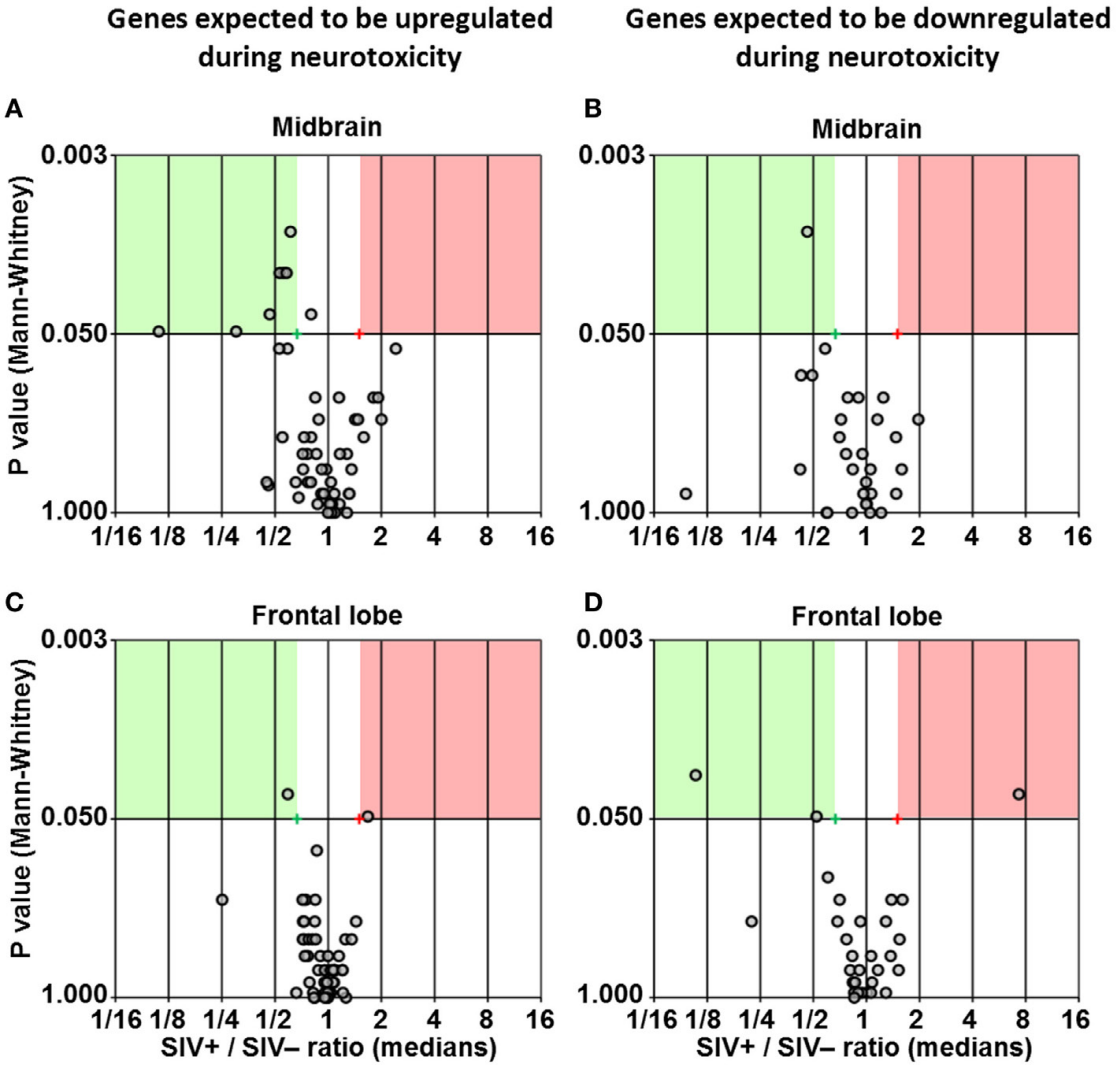
**Differential expression of neurotoxicity genes**. Volcano plots showing the ratio between median gene expression in simian immunodeficiency virus (SIV)-infected macaques and -uninfected macaques (*x* axis; Log_2_ scale), in relation to the *P* value (Mann–Whitney test; *y* axis; Log_20_ scale), for midbrain **(A,B)** and frontal lobe **(C,D)**; each dot represents one gene. Panels **(A,C)** show genes that are expected to be upregulated in neurotoxicity according to the array’s guidelines provided by the manufacturer, whereas panels **(B,D)** show genes that are expected to be downregulated in neurotoxicity according to the same guidelines (see Table S2 in Supplementary Material for details). Red and green areas highlight genes upregulated or downregulated in SIV-infected compared to -uninfected animals (1.5-fold; *P* < 0.05).

**Figure 6:**
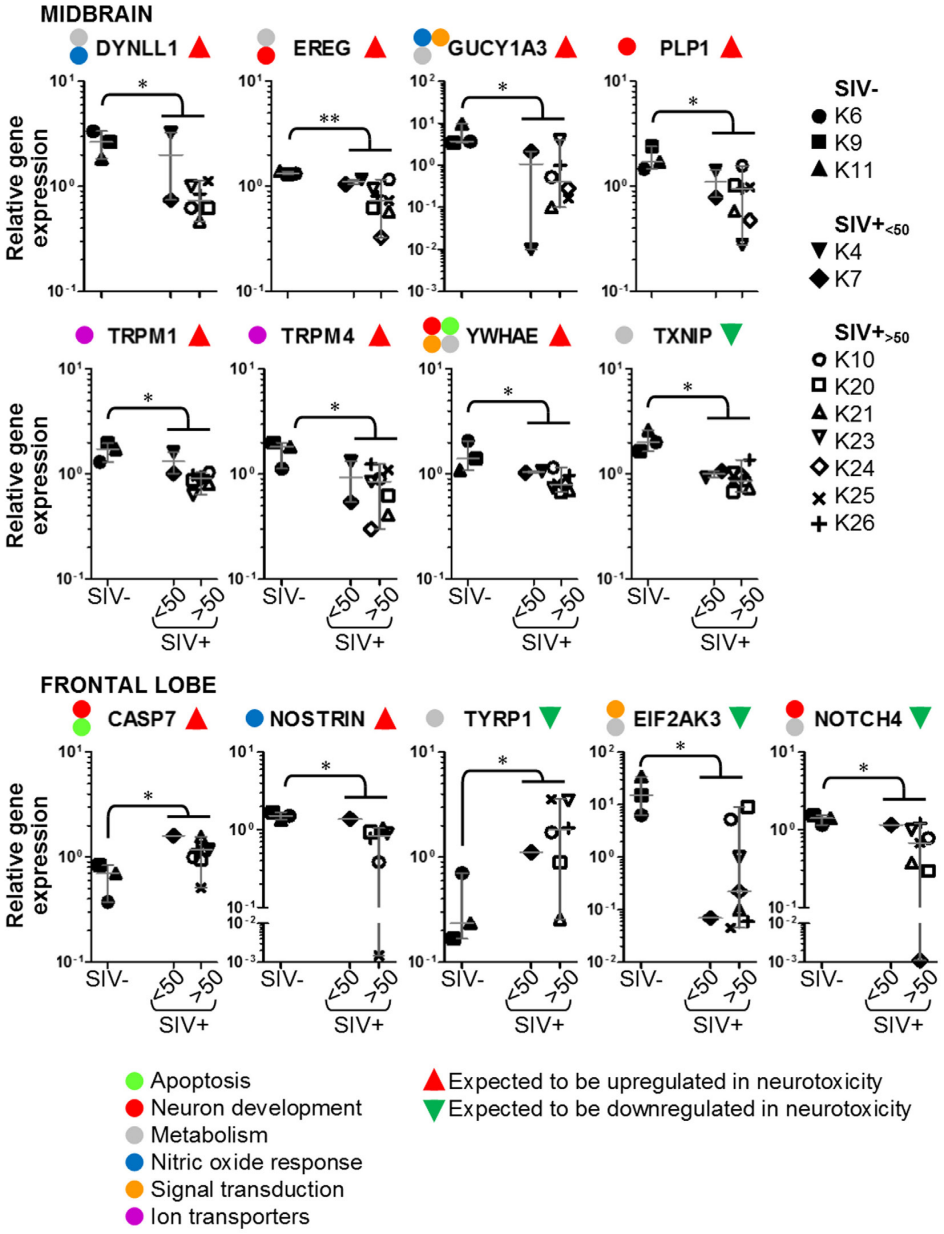
**Details of neurotoxicity genes that are upregulated or downregulated in simian immunodeficiency virus (SIV)-infected macaques**. Plots showing relative expression of the genes in the red and green areas of Figure 5 for uninfected (SIV−), SIV-infected aviraemic (SIV+_<50_), and viremic (SIV+_>50_) animals. All data are normalized against the median for all 12 animals. Each symbol indicates one animal as detailed in the legend; the legend refers to data from both midbrain and frontal lobe; horizontal gray lines within graphs indicate medians, and vertical gray lines show the interquartile ranges (*P < 0.05; **P < 0.01).

We used multigene real-time PCR array in the same samples prepared from midbrain and frontal lobe to analyze the expression of 84 genes, which are either upregulated or downregulated during biological events associated with neurotoxicity (Figures 5 and 6). We did not observe a clear and consistent pattern of gene expression associated with neurotoxicity in either midbrain or frontal lobe. However, subsets of genes associated with neuron development (EREG, PLP1, YWHAE) and nitric oxide response (DYNLL1, GUCY1A3), as well as the ion transporters TRPM1 and TRPM4 were downregulated in the midbrain of SIV-infected compared to -uninfected macaques (Figure 6). The frontal lobe of SIV-infected animals showed upregulation of the apoptotic mediator CASP7, and downregulation of EIF2AK3 and NOTCH4, which have all been associated with neurotoxicity (Figure 6).

### Perivascular Cuffing in Midbrain and Frontal Lobe from SIV-Infected Macaques

**Figure 7:**
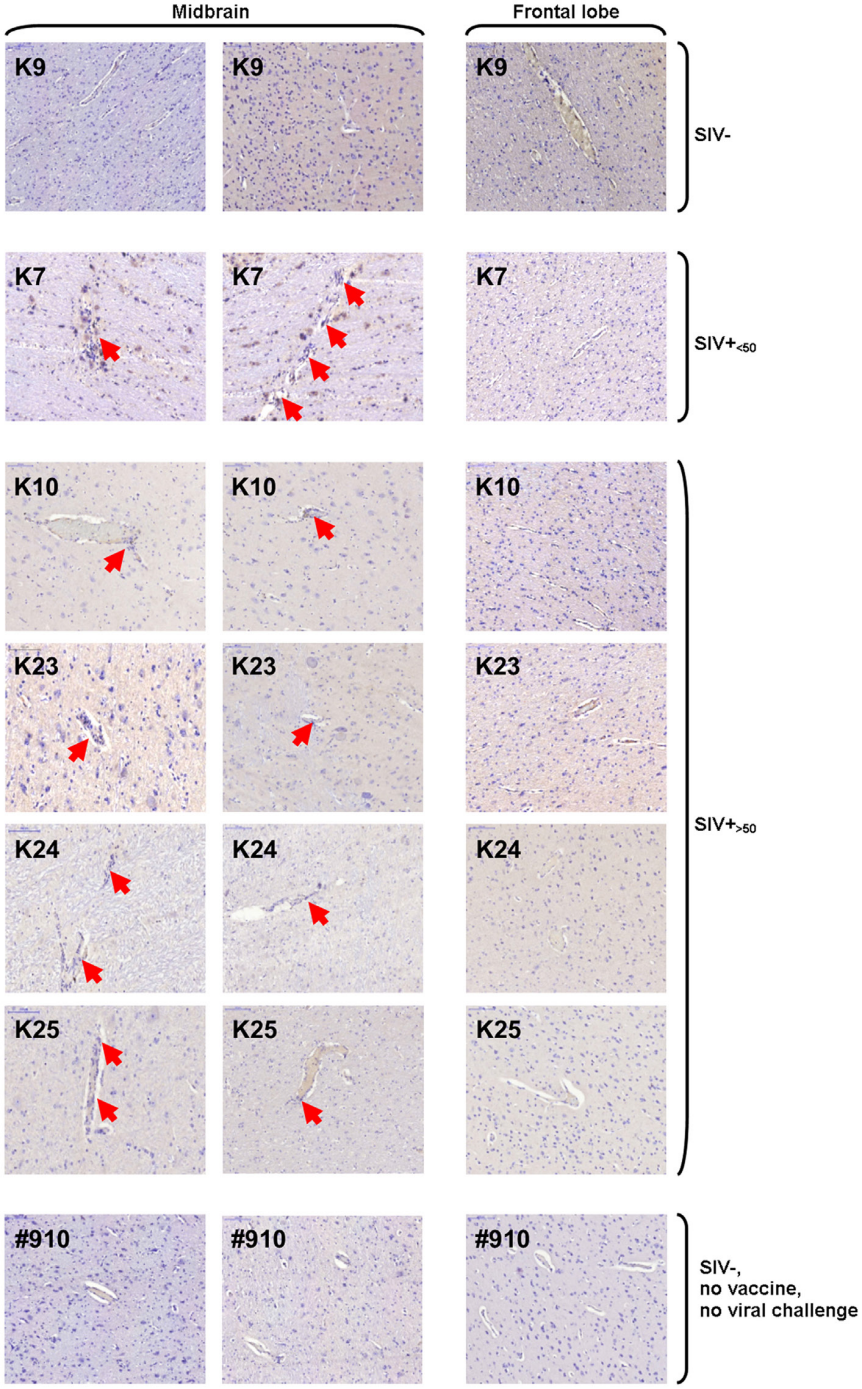
**Hematoxylin staining of midbrain and frontal lobe sections**. Images of one representative frontal lobe and two midbrain sections are shown for one of three uninfected [simian immunodeficiency virus (SIV)−], one of two SIV-infected aviraemic (SIV+_<50_), four of seven SIV-infected viremic (SIV+_>50_) animals, and one example of unvaccinated, non-SIV challenge, healthy macaque (#910). Red arrows indicate sites of perivascular cuffing.

Histologic sections from frontal lobe and midbrain were analyzed for perivascular leukocyte accumulation by standard hematoxylin staining. Evidence of perivascular cuffing was found in midbrain, but not frontal lobe sections of SIV-infected animals, independent of viremia at the time of necropsy (Figure 7). No perivascular lymphocyte accumulation was observed in either midbrain or frontal lobe sections from uninfected macaques (Figure 7). Sections from one representative non-vaccinated, uninfected, non-SIV-challenged macaque (#910) with no signs of perivascular cuffing are shown for comparison.

## Discussion

Human immunodeficiency virus gains access to the CNS *via* infected lymphocytes or macrophages crossing the BBB (14, 15). The evolution of HIV strains segregated to the CNS allows infection of microglial cells and astrocytes (17, 19–22, 40, 41). We found evidence of SIV RNA in the CNS of a fraction of SIV-infected animals, which was not associated with elevated plasma viremia.

Six out of twelve animals in our study were treated with a prophylactic vaccine regimen before SIV challenge. Examples of Adenovector-based vaccines conferring protection from SIV infection or prolonged viremia control have been reported in rhesus macaques challenged with SIVmac251 even at higher doses than the one used in this study, even though the mechanism of protection may depend on either cell-mediated or humoral immunity, depending on the vaccination protocol (42–45). The mechanisms that contributed to the apparent protection offered by the vaccination protocol administered to the animals in this study are still under investigation. However, vaccination did not alter SIV-induced neuroinflammation in animals which became infected. Animals K4, K7, and K10, which became infected despite being administered prophylactic vaccination, showed an inflammatory gene expression profile and perivascular leukocyte infiltration comparable to the six SIV-infected macaques, which were not vaccinated (see Figures 3, 6 and 7). Thus, vaccination may exert a protective effect on SIV infection (animals K6, K9, and K11) or disease progression (animals K4 and K7), but the CNS immune profile of animals K4, K7, and K10 demonstrates that, once infection is established, neuroinflammation is not mitigated by pre-existing, vaccine-induced immunity, and that plasma viremia may not be predictive of immune responses or viral activity in CNS.

Our study shows that a systemic viral insult can have different immunologic effects in anatomically separated regions of the CNS, in that inflammation and immune cell infiltration may be promoted in some sites and suppressed in others. Midbrain from SIV-infected animals showed increased expression of IFN-inducible chemokine genes, the products of which are chemoattractive for both monocytes (CCL13, CXCL11, and CCL1) and activated T lymphocytes (CCL13 and CXCL11). In particular, CXCL11 plays an important role in the recruitment of T cell to the CNS during pathogenic processes (46–48), which is consistent with the observed perivascular cuffing. Because the animals were not perfused prior to brain collection, we cannot exclude that leukocytes trapped in unflushed blood vessels may contribute to the elevated expression of chemokine genes in the midbrain. Nonetheless, the presence of active inflammatory cells in blood vessels deep within the tissue is likely to cause a cascading immune response within the brain itself. We reported a diametrically opposite pattern in the frontal lobe, in which downregulation of inflammatory cytokine and chemokine genes was associated with lack of perivascular accumulation of immune cells. Six of the twelve genes that were downregulated in the frontal lobe of SIV-infected macaques (TNFSF11, CCL2, IL21, IL17A, IL2RA, XCL1) are regulated *via* either STAT1 or STAT3, which are in turn activated *via* IFN-γ and IL-6.

Microglial cells fulfill basic immune functions within the CNS, promoting or preventing infiltration of peripheral leukocytes in the CNS, by differentiating into type 1 or type 2 microglia (49). *In vivo* imaging studies on asymptomatic and ART-treated HIV-infected patients have shown that widespread microglial activation is observed even in patients with undetectable plasma viremia (50). However, our data indicate that the consequences of microglial activation on the immunologic environment in the CNS differ dramatically between midbrain and frontal lobe. The reasons for this differential behavior remain obscure and are a subject of speculation. One possibility is that a predisposition to enhance or suppress immune responses is intrinsic to different regions, and evolutionarily dictated by the sensitivity of a specific site to damage caused by either infectious or inflammatory insults. Thus, it is tempting to speculate that, for the functionality of the CNS, it is critical to protect regions of the mesencephalon and the brain stem from viral pathogenesis, by promoting immune responses and lymphocyte recruitment. Conversely, the frontal lobe or other cerebrum regions may be more resilient to infectious agents, or there may be sufficient functional redundancy to render it more beneficial to suppress immune responses and lymphocyte infiltration, thus limiting the immune-mediated loss of cells in an organ of poor regenerative capacity, at the expense of viral persistence (51). The chronology of antiviral immune responses in the CNS may also differ depending on the anatomical region, spreading in relation to the connection with the choroid plexus, which acts as a critical interface between the CNS and the immune system (52). Because the animals in our study were euthanized soon after the resolution of primary viremia (within 20 weeks from the date of infection), the extent of cell-mediated immune activation may be limited to regions of the brain stem, whereas inflammation and lymphocyte infiltration in the cerebral cortex may be observed only during later, more advanced stages of infection. Nonetheless, the extreme immunologic diversity between frontal lobe and midbrain, suggests that the level of neuronal damage and the mechanisms leading to it may differ between these regions, and that specific areas of the CNS may provide an immune-privileged site for SIV/HIV persistence.

The neurological complications associated with HIV infection vary among patients and depend on disease stage (9). Neuronal damage of the substantia nigra in the midbrain is observed during HIV infection, with negative consequences for the dopaminergic function and subsequent motor dysfunction (53, 54). Reduction of dopamine levels have been observed in the basal ganglia and CSF in HIV-1-infected patients (54, 55), and in the striatum of SIV-infected macaques within 2 months after infection (56), suggesting that damage to this region occurs early during infection. In contrast, prefrontal gray matter atrophy appears to worsen in relation to the duration of infection (57) and is associated with apathy and deficit in verbal learning and memory (58–60). Our data are consistent with the earlier development of neuroinflammation in the midbrain compared to the frontal lobe during the natural course of infection.

The mechanisms that lead to neuronal damage during HIV infection are still unclear. Viral proteins can exert direct neurotoxic activity, and the ensuing inflammatory responses may induce neuronal apoptosis or lead to the accumulation of neurotoxic metabolites (3, 4). The elevated levels of KYN detected in the CSF of animals with high viremia is consistent with the hypothesis that HIV-induced neuroinflammation may lead to the production of neurotoxic catabolites of the KYN pathway, such as the NMDA agonist QA (39). We found downregulation of YWHAE expression in the midbrain of SIV-infected macaques. Although YWHAE is one of the genes which we expected to be upregulated during neurotxicity, based on the definition of the gene array, the protein encoded by YWHAE (14-3-3ε protein) is involved in the inhibition of apoptosis *via* association with Bcl-2, and CSF levels of YWHAE are reduced in HIV-infected patients who display cognitive impairment (61). The gene expression pattern in the frontal lobe was consistent with the activation of pathways leading to neuronal apoptosis (downregulation of EIF2AK3 and NOTCH4 and upregulation of CASP7) in SIV-infected macaques.

The main obstacle to HIV eradication is the establishment of reservoirs of infected cells, which persist despite ART and are not susceptible to immune-mediated clearance (62). Novel immunotherapeutic strategies are being considered and tested at preclinical and clinical level to enhance and redirect antiviral T cell responses against latent reservoirs (62). The presence of proviral DNA in brain-infiltrating macrophages, microglia, and astrocytes (18, 29, 31, 40), indicates that the CNS harbors a population of productively infected cells. Our data show that viral RNA can be detected in midbrain and frontal lobe and cannot be predicted by plasma viremia. The local downregulation and suppression of chemokine genes raise the possibility that provirus-bearing cells in the frontal lobe may be shielded from T cell-mediated immunity. Thus, even when enhanced by immunotherapeutic approaches, HIV-specific T lymphocytes may need to overcome an immunoregulatory barrier to reach and clear infected cells in certain regions of the CNS.

Human immunodeficiency virus is an archetypal example of a systemic infection which can gain access to the CNS and result in profound multi organ, immune-mediated pathogenesis. The complex interactions between virus, immune system, and CNS underlie both the development of neurological symptoms and the perpetuation of a local viral reservoir. The data presented in this study offer a new perspective on how immune responses are modulated in different regions of the CNS in the simian model of HIV infection. Our findings highlight the possibility that specific anatomical locations may provide a uniquely privileged environment in which HIV may remain inaccessible to cell-mediated antiviral immunity, a potentially critical hurdle in the search for a cure.

## Author Contributions

AB and DeF designed the study, analyzed, and interpreted data. SP and NA contributed to the study, design, and data interpretations. BT, CR, DiF, NB, and CH acquired, analyzed, and interpreted data. MK, VT, and GH analyzed and interpreted data. All authors contributed to the drafting and revising of the manuscript and have approved the final version. All authors agreed to be accountable for all aspects of the work in ensuring that questions related to the accuracy or integrity of any part of the work are appropriately investigated and resolved.

## Conflict of Interest Statement

The authors declare that the research was conducted in the absence of any commercial or financial relationships that could be construed as a potential conflict of interest.
