# Supplementary material for: Immune Responses in the Central Nervous System Are Anatomically Segregated in a Non-Human Primate Model of Human Immunodeficiency Virus Infection
Source: Front Immunol. 2017 Mar 30;8:361. doi: 10.3389/fimmu.2017.00361 (PMC5371826; doi:10.3389/fimmu.2017.00361)
Supplement: Supplementary file 1 [file table_1.docx]

**Table S1. List of genes in the Qiagen RT^2^ profiler gene array PAQQ-011Z.**

| Chemokines | CCL1 (I-309); CCL11 (eotaxin); CCL13 (MCP-4); CCL17 (TARC); CCL2 (MCP-1); CCL20 (MIP-3A); CCL23 (MPIF-1); CCL5 (RANTES); CX3CL1; CXCL11 (I-TAC; IP-9); CXCL12 (SDF1); CXCL13; CXCL6 (GCP-2); CXCL9; LOC714751 (CCL7); CCL26; XCL1 |
| --- | --- |
| Chemokine Receptors | CCL13 (MCP-4); CCR1; CCR3; CCR4; CCR6; CCR8; CX3CR1; CXCR1 (IL8RA); CXCR4 |
| Interleukins | IL12B; IL13; IL15; IL16; IL17A; IL17F; IL1A; IL1B; IL1RN; IL2; IL20; IL21; IL3; IL4; IL5; IL6; IL7; IL8; LOC710618 (IL17C); IL9 |
| Interleukin Receptors | IL10RA; IL10RB; IL11RA; IL12RB1; IL12RB2; IL15RA; IL1R1; IL1R2; IL2RA (CD25); IL2RB; IL2RG; IL5RA (CD125); IL6R; IL8RB; IL9R; IL5RA |
| Other Cytokines | AIMP1 (SCYE1); BMP2; CD40LG (TNFSF5); CD70 (TNFSF7); CSF1 (MCSF); CSF2 (GM-CSF); FASLG (TNFSF6); IFNA2; IFNG; LTA (TNFB); MIF; NAMPT; OSM; PF4; SPP1; TNF; TNFSF10 (TRAIL); TNFSF11; TNFSF13B; TNFSF14; TNFSF4(OX40L); VEGFA |
| Other Cytokine Receptors | TNFRSF11B (OPG) |
