# Supplementary material for: Immune Responses in the Central Nervous System Are Anatomically Segregated in a Non-Human Primate Model of Human Immunodeficiency Virus Infection
Source: Front Immunol. 2017 Mar 30;8:361. doi: 10.3389/fimmu.2017.00361 (PMC5371826; doi:10.3389/fimmu.2017.00361)
Supplement: Supplementary file 2 [file table_2.docx]

**Table S2. List of genes in the Qiagen RT^2^ profiler gene array PAQQ-069Z.**

| *Genes expected to be upregulated in neurotoxicity* | |
| --- | --- |
| Apoptosis | ABL1; BAG4; BIK; CASP7; CDKN1A (p21CIP1; WAF1); CIDEA; CIDEB; CRADD; DAPK1; FAS (TNFRSF6); FASLG (TNFSF6); GCH1; SOD2; TNFRSF10B (DR5); TNFRSF11B (OPG); TP53 (p53); TRAF4; YWHAE |
| Neuron Development | CASP7; DRP2; EREG; HEPH; IL10; MMP9; PLP1; SOD2; TP53 (p53); YWHAE |
| Metabolism  (Regulation & Mechanism): | ABL1; ARG2; ARRB1; ATF4; CDKN1A (p21CIP1; WAF1); CIDEA; DDIT3 (GADD153; CHOP); DYNLL1; EREG; GAL; GUCY1A3; HSP90AA1; IL10; NFKB1; POU1F1 (Pit-1); RASD1; SLC16A3; SOD2; TNFRSF10B (DR5); TP53 (p53); TRAF2; USP7; YWHAE |
| Nitric Oxide Response | ARG1; ARG2; DYNLL1; GUCY1A3; NOS1AP; NOSIP; NOSTRIN; RASD1; SLC16A3 |
| Signal Transduction | ABL1; ARRB1; CD8B; CDKN1A (p21CIP1; WAF1); CIDEA; CIDEB; DAPK1; DDIT3 (GADD153; CHOP); FASLG (TNFSF6); GPR37; GUCY1A3; HSPA5 (GRP78); IL10; RASD1; TNFRSF10B (DR5); TP53 (p53); TRAF2; YWHAE |
| Ion Transporters | CAMK2D; CLCN5; CLCNKA; HEPH; SFXN5; TRPM1; TRPM4 |
| Other Genes | ANGPTL4; PAPPA; PDIA4 |
| *Genes expected to be downregulated in neurotoxicity* | |
| Apoptosis | LTA (TNFB); NOL3; TNFRSF25 (DR3); XIAP (BIRC4) |
| Neuron Development | BDNF; KIT (CD117); LEFTY2 (EBAF); LTA (TNFB); NOTCH4; SEMA3B. |
| Metabolism  (Regulation & Mechanism): | BDNF; CAMK2A; CCND1; CDO1; DRD2; EIF2AK3; GALC; GRIN1; GSR; HTR1A; KIT (CD117); LDHA; NOTCH4; PRIM2; TPH1; TXNIP; TYRP1. |
| Signal Transduction | BIRC2 (c-IAP1); CCND1; DRD2; EIF2AK3; GSN; HTR1A; KIT (CD117); TACR1 |
| Ion Transporters | DRD2; GRIN1 |
| Other Genes | COL12A1; EGLN3; HTR3A; NUP50. |
