# Supplementary material for: Immune Responses in the Central Nervous System Are Anatomically Segregated in a Non-Human Primate Model of Human Immunodeficiency Virus Infection
Source: Front Immunol. 2017 Mar 30;8:361. doi: 10.3389/fimmu.2017.00361 (PMC5371826; doi:10.3389/fimmu.2017.00361)
Supplement: Supplementary file 3 [file table_3.docx]

**Table S3. Primer sequences for real time PCR assay.**

| Gene | Forward primer | Reverse primer |
| --- | --- | --- |
| GAPDH | GTCTGGAAAAACCTGCCAAG | ACCTGGTGCTCAGTGTAGCC |
| FoxP3 | GCTGGAGAAGGAGAAGCTGA | CACAGATGAAGCCTTGGTCA |
| CTLA-4 | ATCTGCAAGGTGGAGCTCAT | AATCTGGGTTCCATTGCCTA |
| IDO | ACAGAATGCTGGTGGAGGAC | GGAAGTTCCTGTGAGCTGGT |
| IL-10 | GAGAACCACGACCCAGACAT | CAGCCTGAGGGTCTTCAGAT |
| TGF-β | GGGACTATCCACCTGCAAGA | CAGCTTGGACAGGATCTGG |
